# Superior Vena Cava Syndrome: An Umbrella Review

**DOI:** 10.7759/cureus.42227

**Published:** 2023-07-20

**Authors:** Rajendra P Shah, Olayiwola Bolaji, Sanchit Duhan, Anderson C Ariaga, Bijeta Keisham, Timir Paul, Wael Aljaroudi, M. Chadi Alraies

**Affiliations:** 1 Department of Internal Medicine, Vassar Brothers Medical Center, Poughkeepsie, USA; 2 Department of Internal Medicine, University of Maryland Capital Regional Medical Center, Largo, USA; 3 Department of Internal Medicine, Sinai Hospital of Baltimore, Baltimore, USA; 4 Sinai Center for Thrombosis and Research, Sinai Hospital of Baltimore, Baltimore, USA; 5 Section of Interventional Cardiology, University of Tennessee at Nashville/Ascension Saint Thomas Hospital, Nashville, USA; 6 Department of Cardiology, Augusta University Medical College of Georgia, Augusta, USA; 7 Department of Cardiology, Detroit Medical Center, Detroit, USA

**Keywords:** superior vena cava (svc), cerebral venous thrombosis (cvt), endovascular stent therapy, mediastinitis, superior vena cava syndrome

## Abstract

Superior vena cava syndrome (SVCS) is a medical emergency that encompasses an array of signs and symptoms due to obstruction of blood flow through the superior vena cava (SVC). It poses a significant healthcare burden due to its associated morbidity and mortality. Its impact on the healthcare system continues to grow due to the increasing incidence of the condition. This incidence trend has been attributed to the growing use of catheters, pacemakers, and defibrillators, although it is a rare complication of these devices. The most common cause of SVCS remains malignancies accounting for up to 60% of the cases. Understanding the pathophysiology of SVCS requires understanding the anatomy, the SVC drains blood from the right and left brachiocephalic veins, which drain the head and the upper extremities accounting for about one-third of the venous blood to the heart. The most common presenting symptoms of SVCS are swelling of the face and hand, chest pain, respiratory symptoms (dyspnea, stridor, cough, hoarseness, and dysphagia), and neurologic manifestations (headaches, confusion, or visual/auditory disturbances). Symptoms generally worsen in a supine position. Diagnosis typically requires imaging, and SVCS can be graded based on classification schemas depending on the severity of symptoms and the location, understanding, and degree of obstruction. Over the past decades, the management modalities of SVCS have evolved to meet the increasing burden of the condition. Here, we present an umbrella review providing an overall assessment of the available information on SVCS, including the various management options, their indications, and a comparison of the advantages and disadvantages of these modalities.

## Introduction and background

Superior vena cava syndrome (SVCS) is a clinical condition that comprises a spectrum of signs and symptoms due to obstruction of blood flow through the superior vena cava (SVC) [1]. The first described case of SVCS was a patient with a syphilitic aortic aneurysm in 1757. In a review published in 1954, from the 274 well-documented cases reviewed by Schecter, about 40% were due to a syphilitic aneurysm or tuberculous mediastinitis [2]; this study was significant at the time as it showed that SVCS had other etiologies other than syphilitic aortic aneurysms. It is estimated that malignant tumors account for 60% of cases, while iatrogenic causes from thrombosis or stenosis caused by central lines or medical devices account for 30-40% [3]. This review article provides a concise, evidence-based review of the management of SVCS.

The incidence of SVCS continues to rise due to the increasing use of catheters, pacemakers, and defibrillators [4]. Rice et al. found that 28% of all SVCS is associated with a device or catheter [5]. While complications arising from these devices contribute to a significant proportion of SVCS cases, Chee et al. observed that it is a rare complication affecting only about 0.1-3.3% of all pacemaker patients [4]. Major thrombophilia and Behcet’s disease are also common causes of spontaneous SVCS. In older adults, malignancy is the most common cause of SVCS [6-8].

Understanding the pathophysiology of SVCS requires an understanding of the anatomy. The SVC drains about one-third of the venous blood to the heart. It receives venous blood from the right and left brachiocephalic veins, which drain the head and the upper extremities. In a computed tomography (CT) scan, the length of the SVC is 7.1 cm ± 1.4 with a diameter of 2.1 cm ± 0.7 (which varies with volume) [9]. A cross-sectional diameter of < 1.07 cm^2^ indicates SVC obstruction or compression [10].

The most common presenting symptoms of SVCS are swelling of the face and hand with distension of vessels in the subcutaneous tissue, cyanosis or plethora, chest pain, respiratory symptoms due to edema, and swelling of parts of the respiratory tract, including the pharynx and larynx (dyspnea, stridor, cough, hoarseness, and dysphagia), and neurologic manifestations (headaches, confusion, or visual/auditory disturbances), enlargement of subcutaneous vessels, and edema of the arms, head, and neck. Cardiac function compromise can also occur due to mass effect on the heart or impaired venous return due to SVC obstruction [11]. Symptoms generally worsen in a supine position. Other signs and symptoms specific to the causative agent may also be observed.

## Review

Classification and scoring system for SVCS

**Figure 1:**
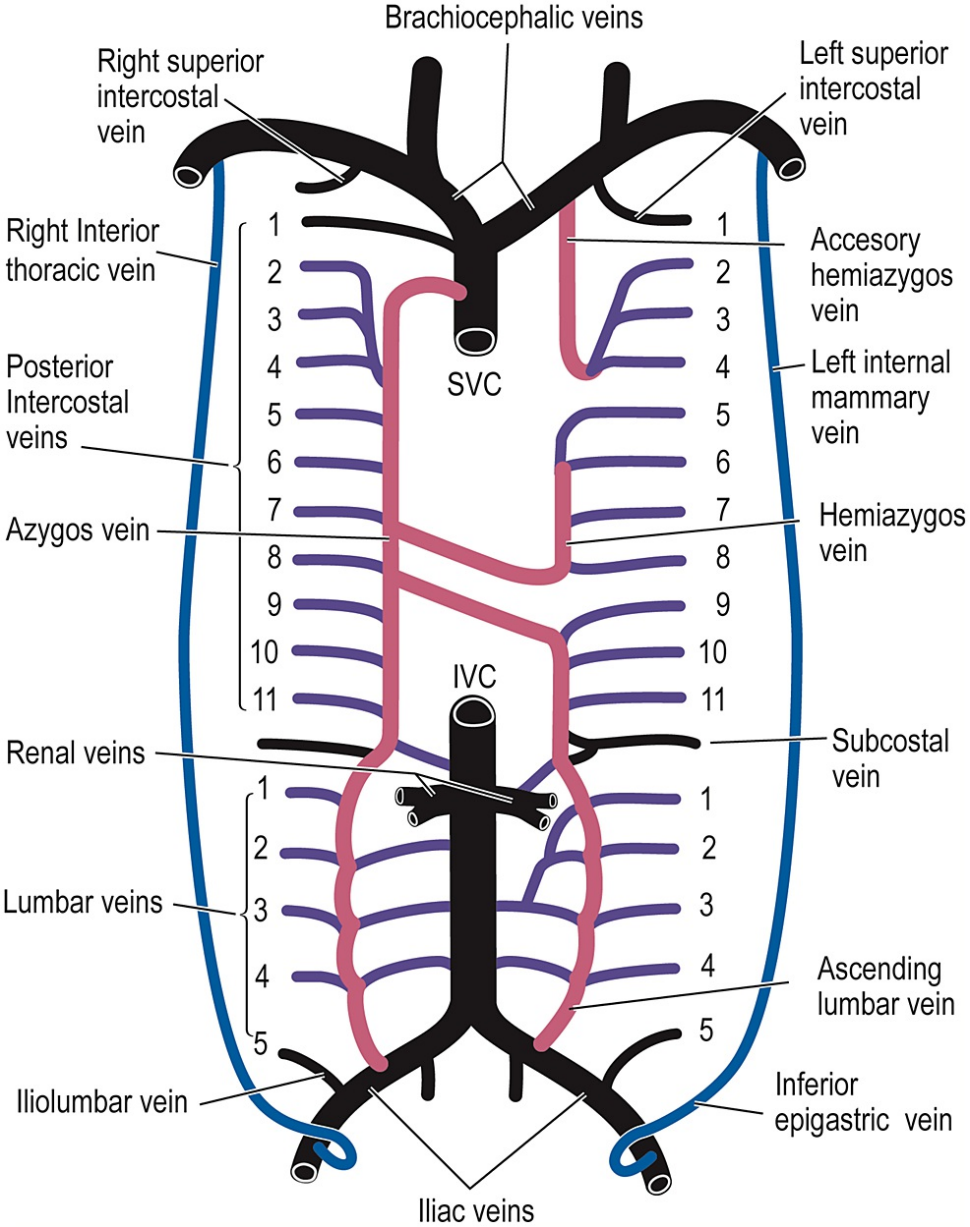
Collateral circulation in SVCS Picture credits: Azizi et al., 2020 [1]. Publication license obtained from Elsevier. SVCS: superior vena cava syndrome; IVC: inferior vena cava

The severity of symptoms directly correlates to the understanding and extent of the venous obstruction and inversely to the development of venous collaterals [11,12]. Four main collateral pathways can develop with SVCS, with the essential collateral pathway being the azygos-hemiazygos pathway (via the intercostal and lumbar veins). The other collateral pathways that might be created are the internal thoracic route (via the epigastric veins and superficial thoracic veins), the lateral thoracic route (via the superficial circumflex and long saphenous and femoral vein), and the vertebral and paravertebral route (Figure 1). In a very severe and rare form of SVCS, the hot quadrate sign-pathway can connect the superior epigastric and internal thoracic veins [11,13,14].

Obstruction of the SVC can occur at various levels. The most common landmark used to describe position is the azygos vein. Blocks can be proximal, distal, or at the level of the azygos vein. In obstructions above the azygos, blood can still return to the SVC and eventually the heart through the azygos vein [15]. Collaterals between the intercostals and azygos veins maintain blood flow into the terminal portion of the SVC. On the other hand, blockages below the azygos vein prevent blood flow from the SVC into the right atrium. As a result, collaterals between the azygos and other veins allow for retrograde flow and eventual blood return through the inferior vena cava (IVC) [15].

Just like obstructions below the azygos, blockages at the level of the azygos vein tend to produce severe symptoms, as in both cases, there are no means of obtaining flow through the SVC. In this case, blood cannot return to the azygos system, forcing blood through the collaterals to the IVC [15]. Other than the level of obstruction, another factor affecting symptomatology is the acuity of obstruction. According to Yu et al., a patient can present without clinical symptoms, likely from a slow-growing tumor or acutely due to acute thrombosis resulting in complete obstruction of the SVC [11]. The various classification and triage schemas for SVC obstruction try to incorporate these factors.

Though no standardized classification system is currently used for SVC obstruction, the Stanford method is the most commonly used, especially in patients with severe symptoms, because it helps identify patients at risk for respiratory or cerebral compromise requiring urgent intervention. It classifies SVC obstruction by venography [12]. Yu et al. proposed a grading system that allows clinicians to differentiate between severe, life-threatening, and non-life-threatening clinical conditions. This system categorizes patients mainly based on the severity of symptoms which generally correlates to the degree of obstruction [11].

Another scoring system is the Kishi scoring system which assesses the need for intervention; a score of ≥4 suggests a need for percutaneous stent placement, while a score of <4 suggests conservative management after considering the cause of obstruction, alternative management, and the patient's overall prognosis. The scoring system is also primarily based on the severity of presenting symptoms. The most recent classification system proposed by Azizi et al. is based on anatomical location and severity of SVC obstruction. This new classification is believed to help guide management and facilitate clinician communication more efficiently than the other classification systems [1].

Diagnostic approach

Diagnosis of SVCS involves a combination of clinical signs and symptoms and various imaging modalities that can help confirm the diagnosis and provide more information, such as the degree of obstruction and an idea of the possible etiology. With advancements in medicine and technology, different imaging modalities have been made available for diagnosing SVCS. Frequently used imaging modalities include plain radiography, duplex ultrasound, contrast-enhanced CT or magnetic resonance imaging (MRI), conventional catheter-based digital subtraction venography, and magnetic resonance venography. Plain radiography can help provide details about a mass, tumor, or pleural effusion that might cause SVC obstruction but often must be followed by more definitive diagnostic imaging. Another option is Duplex ultrasonography (US) which can help detect the presence of any thrombus within jugular, subclavian, and axillary veins. It is also helpful to see device-associated thrombi formation associated with pacemakers and catheters in SVCS. The major drawback of Duplex US is that it cannot visualize the SVC adequately because of the ribs and lung shadows [16]. Contrast-enhanced CT is the gold standard choice of imaging in emergent cases, with high sensitivity (96%) and specificity (92%) [9,17]. It can produce a detailed image of the SVC and elucidate the presence of intrinsic or extrinsic obstruction, extent of blockage, and presence of collateral circulation. In contrast-enhanced CT, the presence of collateral circulation is a good prognostic factor for symptomatic SVCS as it implies some degree of adaptation by the body to allow venous return to the heart. Still, it is essential to note that collateral pathways remain even after percutaneous intervention and improvement of symptoms [18]. In patients allergic to contrast dye, contrast-enhanced MRI is a good substitute. Digital subtraction venography is the gold standard for diagnosing SVC obstruction and SVC thrombus, especially in non-emergent cases. It can help delineate the presence of thrombus and collateral pathways and define the severity. The major drawback is the inability to identify extrinsic causes of SVC obstruction. Also, MRI venography is a good substitute [16,19]. Since most SVCS is due to malignancies, it is essential to establish a definitive diagnosis to manage SVCS patients appropriately. Establishing a diagnosis might involve further workup with sputum cytology, pleural puncture with fluid cytology, and image-guided needle biopsy.

Management of SVCS

**Figure 2:**
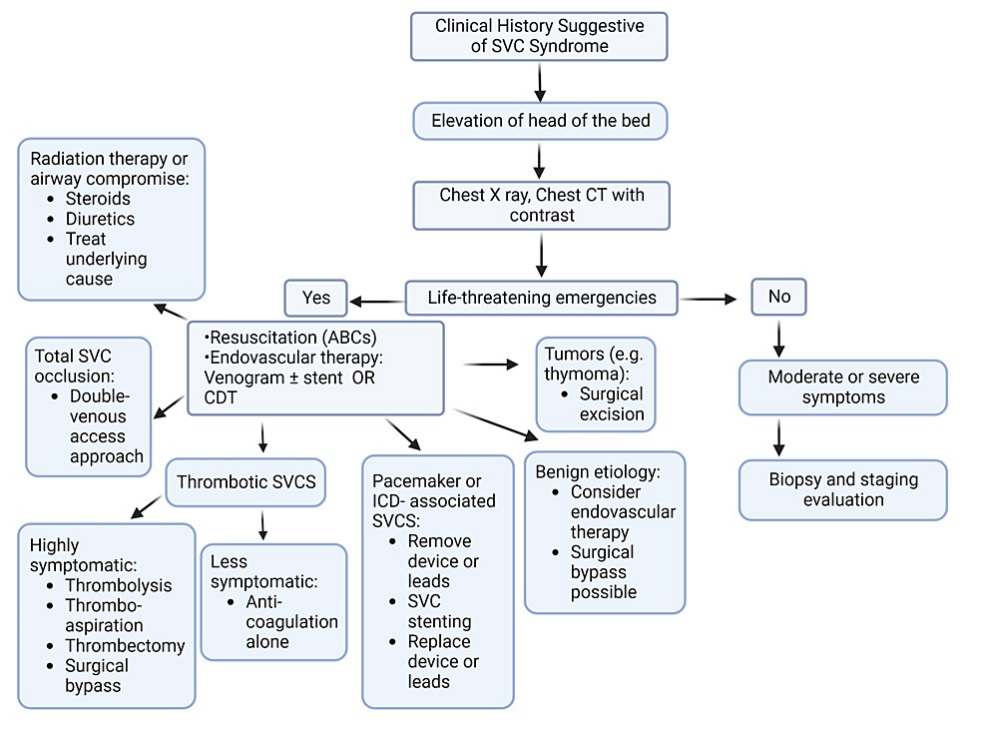
Management algorithm for SVCS ABCs: airway, breathing, circulation; CT: computed tomography; SVCS: superior vena cava syndrome; CTD: catheter-directed thrombolysis; ICD: implantable cardioverter-defibrillator Image credits: Author "Sanchit Duhan." Created using Biorender.com.

Management of SVCS involves a multidisciplinary team approach. The team should include pulmonology, oncology, cardiology, vascular, endovascular, surgery specialists, and radiology. The emphasis of therapy lies in identifying the etiology and targeted management as well as symptom control. Management should also be tailored around the severity of symptoms. The outcome heavily depends on the acuity and severity of obstruction and the underlying etiology, with some patients achieving long-term relapse. At the same time, some patients can attain complete resolution. Cardiopulmonary resuscitation is followed by endovascular therapy (venogram and recanalization with or without stenting or catheter-directed thrombolysis (CDT)) in life-threatening situations. General supportive measures include elevation of the head of the bed to reduce the hydrostatic pressure in the head and neck. The use of steroids and diuretics are common initial practice. Although there is not much data supporting their efficacy in life-threatening cases, the literature supports their effectiveness in patients undergoing radiation therapy (RT) or with airway compromise as it reduces the development and extension of edema [20-22]. For malignancy which is the most common cause, current treatment options include chemotherapy with or without RT, surgical bypass, or endovascular therapy (including angioplasty, stenting, and catheter-based thrombus removal) (Figure 2) [1].

One of the mainstays of management is endovascular therapy. The first endovascular stent for SVCS was described in 1986 by Charnsangavej et al. Since then, it has continued to gain popularity and is currently the standard of care for both benign and malignant SVCS [23]. It benefits those presenting with life-threatening symptoms as it provides rapid resolution without affecting the need for a definitive diagnosis. It can be combined with other modalities of management to achieve better outcomes. Observational studies suggest that more than 90% of patients have symptomatic relief with a technical success rate of about 80-98%, a restenosis rate of 4.3-29.5%, and a recurrence rate of 1.2-20.5% [1,6,24-28]. Some indications for endovascular stenting include patients with life-threatening symptoms, symptomatic patients without RT or chemotherapy, and patients with contraindications to RT or chemotherapy [11].

Endovascular stenting is generally performed under local anesthesia with conscious sedation. The patient is placed in a supine position with the head of the bed elevated with or without extending the torso. General anesthesia may be required if lying flat results in clinically significant desaturation due to airway compromise or edema [29,30]. Access to the vena cava is usually through single or double ultrasound-guided venous access, depending on the size and extent of the occlusion. A single US-guided femoral access is sufficient for a non-occlusive small lesion. Still, other access through the upper extremities (such as the basilic, brachial, and axillary veins) and jugular access can provide good images and allow easy device deployment [19,31,32]. However, most interventionists use two venous accesses to allow for better imaging. Using two different venous accesses is advised in patients with total occlusion of the SVC for successful recanalization [30]. The rate of complications is acceptably low, mitigated by the experience of the performing physician. The cumulative incidence of complications from endovascular therapy is < 8%, according to Rachapalli et al. [30], and the procedural mortality rate is about 2% [33]. Minor complications include hematoma and local infections at the puncture site. In contrast, significant complications include sinus arrest, pericardial tamponade (0.1-1.8%), SVC rupture, stent migration, in-stent restenosis, pulmonary edema, pulmonary embolism, and cardiac injury.

SVCS caused by thrombosis can be managed by CDT or thrombo-aspiration, and in highly symptomatic patients, thrombectomy should be offered. Mildly symptomatic patients and patients with incomplete thrombosis can be managed conservatively with anticoagulation therapy alone [34-36]. While there is no guideline or consensus on managing a patient with SVCS associated with a pacemaker or defibrillator, it is generally advisable to avoid stent placement without removing the device or lead that might have precipitated the thrombosis [37]. Klop and colleagues reported that most interventionists prefer lead retraction and placement of new leads immediately after SVC stenting, while few prefer stent placement without lead retraction [37,38].

Another crucial part of the management of SVCS is RT. Before advancements in endovascular treatment, RT was the first-choice therapy as it was believed to be the fastest means of symptomatic relief. However, recent studies have shown that about 80% of patients achieved symptomatic relief, with relief taking up to three days to four weeks. Although RT reduces tumor burden, it does provide a challenge as performing histologic diagnosis once the patient has undergone RT is challenging.

Surgical intervention is another pillar of SVCS management. An open surgical bypass with a reconstruction of the SVC can be offered for patients with extensive venous thrombosis or highly symptomatic occlusion. The bypass is usually performed from the innominate or jugular vein to the right atrial appendage or the SVC using the saphenous vein graft [39]. It is worth noting that about 50% of patients who underwent bypass surgery further need endovascular stenting to maintain secondary patency.

Anticoagulation should be continued for at least three months in patients with thrombosis, especially in device-associated/induced thrombosis or primary thrombosis. Still, currently, no data support the efficacy of the continuation of antiplatelet or anticoagulant in preventing the recurrence of thrombosis after successful intervention [40,41]. Table 1 describes the advantages and disadvantages of the different treatment modalities.

**Table 1:** Treatment modalities of SVCS NSCLC: non-small cell lung cancer, SVC: superior vena cava, SVCS: superior vena cava syndrome, SCLC: small cell lung cancer Credits: Author "Olayiwola Bolaji."

	Radiation [41]	Stent [41]	Surgery [39]	Chemotherapy [41]
Chance of symptom relief (%)	56-96%	80-95%	79-93%	59-77%
Time for symptom relief	3-30 days	0-72 hours	0-72 hours	1-2 weeks
Advantages	Decreases tumor burden	Minimally invasive with a high technical success rate	Provides durable reconstruction of SVC	First-line therapy for chemo-sensitive malignancies (e.g., SCLC, non-Hodgkin's lymphoma, germ cell tumor)
		Immediate resolution of symptoms		
	Definitive therapy in patients with stage II/III NSCLC, low-grade lymphoma	It allows for histologic diagnosis	High rate of graft patency	
		It can be combined with other modalities, including surgery, radiation, and chemotherapy.		
	Very well-studied modality	Short post-procedure course	Could be considered in SVC syndrome from benign etiology and patients with a long life expectancy	
Disadvantages	Alters histologic diagnosis	Significant complications are less common than surgery but include stent migration, stent re-occlusion, and pericardial tamponade.	Invasive surgery	Treatment roles in other etiologies are not well established.
	Delayed relief of symptoms (3-30 days)		It can require prolonged mechanical ventilation and possibly a tracheostomy.	Delayed relief of symptoms
	Multiple well-known complications such as SVC perforation, inhibition of venous collateral development, and fibrotic changes in blood vessels from radiation.	Lower overall durability than surgery, making it less ideal in patients with a long life expectancy.	Higher rates of complications compared to endovascular therapy include mediastinal hematoma, pulmonary embolism, and deep vein thrombosis.	Complications include gastrointestinal disturbance, toxicity of chemotherapeutic agents, including anemia, neutropenia, infections, and coagulopathy.
	Daily treatments are not practical and affect the quality of life.		Higher procedure-related morbidity	

## Conclusions

SVCS is a complex and challenging condition with severe outcomes if not promptly diagnosed and managed appropriately. The most common cause remains malignancy, but the incidence of non-malignant SVCS is steadily increasing. Advancement in percutaneous therapy has shifted management modalities from RT to endovascular treatment with significant favorable outcomes, although no standardized guideline recommendations are yet. Further research and multicenter studies are needed to standardize the classification system, management modalities, and use of anticoagulants. It also provides an avenue for possible clinical trials comparing various venous stents.

## Figures and Tables

**Figure 3:**
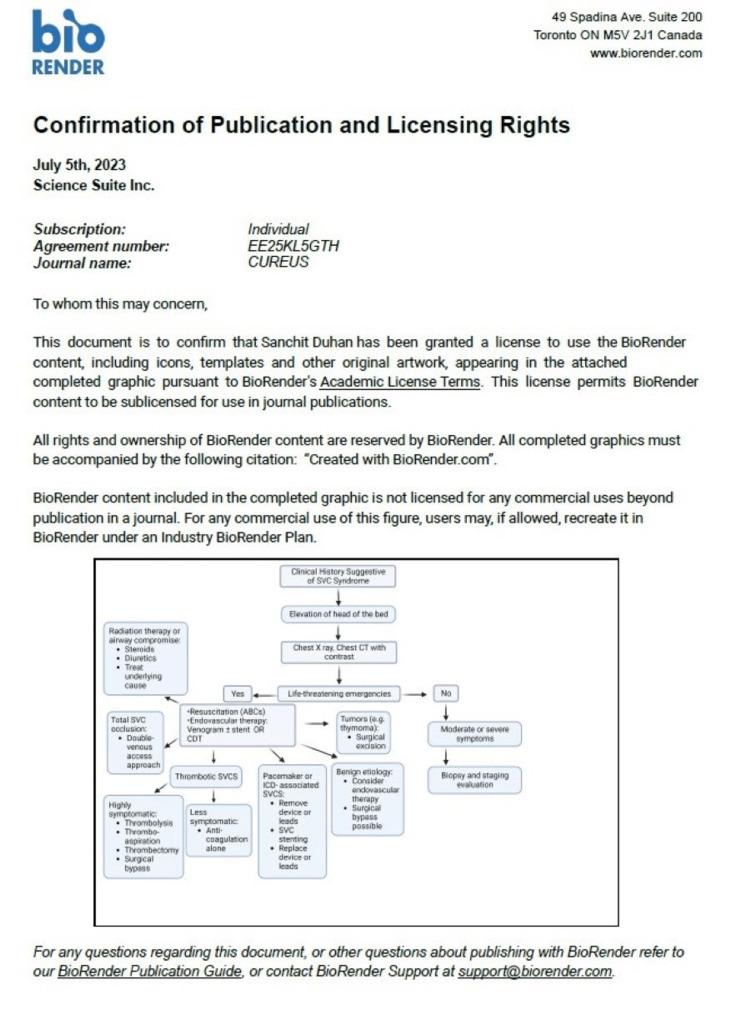
Publication license for Figure 2 Created using Biorender.com

## Appendices

Appropriate permission has been obtained from Elsevier for Figure 1 and cited in the legend. Figure 2 was created with Biorender.com, and the authors also obtained a publication license (see Figure 3).
